# Trends in the leading causes of childhood mortality from 2004 to 2016 in Qatar

**DOI:** 10.5195/cajgh.2018.334

**Published:** 2018-11-01

**Authors:** Mohammed Al-Thani, Al-Anoud Al-Thani, Amine Toumi, ShamsEldin Khalifa, Muhammad Asif Ijaz, Hammad Akram

**Affiliations:** Ministry of Public Health, State of Qatar

**Keywords:** Childhood mortality, Qatar, Childhood mortality causes, Sex ratio, Public Health, Epidemiology

## Abstract

**Introduction:**

Childhood mortality is an important health indicator that reflects the overall health status of a population. Despite the decrease in global childhood mortality rates over the past decades, it still remains an important public health issue in Qatar.

**Methods:**

The data from 2004–2016 were extracted from the Qatar Ministry of Public Health Birth and Death Database. International Classification of Diseases (ICD-10) was used for coding the causes of death. The childhood mortality rate was defined as the probability of a child dying between the first and the fifth birthday, expressed as the number of deaths per 1,000 children surviving to 12 months of age. The sex ratio was calculated by dividing the mortality rate of males by that of females. Mann-Kendall trend test was performed to examine time trends. Relative risks were calculated to examine differences by nationality (Qatari and non-Qatari) and sex.

**Results:**

A significant decrease in mortality rate of children aged one to five was observed from 1.76 to 1.05 per 1000 children between 2004 and 2016 (Kendall tau=−0.6, p=0.004). Three prominent causes of mortality were motor vehicle accidents, congenital malformations of the circulatory system, and accidental drowning/submersion. A statistically non-significant decrease in childhood mortality from motor vehicle accidents was oberved for all nationalities (total (Kendall tau=−0.03), Qatari (Kendall tau=−0.14), and non-Qatari (Kendall tau=−0.12)). A significant decrease was seen for total accidental drowning and submersion (Kendall tau=−0.54, p=0.012), while no statistically significant decrease was seen for total congenital malformations of the circulatory system (Kendall tau=−0.36, NS). The Qatari population did have a significant decrease in childhood mortality due to congenital malformations of the circulatory system (Kendall tau=−0.67, p=0.003) and accidental drowning and submersion (Kendall tau=−0.55, p=0.016).

**Conclusion:**

The study is a first attempt to evaluate childhood mortality statistics from Qatar and could be useful in supporting Qatar’s ongoing national health strategy programs.

## Introduction

The mortality rate for children under the age of five is defined as the number of deaths before the age of five per 1,000 live births in a region of interest and is a commonly used health indicator.^1^ Decline in under-five mortality is a global trend reaching 58% reduction from 1990 to 2017.^2^ However, under five mortality is still high among countries in the World Health Organization (WHO) African region, reaching 100 deaths per 1,000 live births in some countries.^2^,^3^ It is an important health indicator that represents the health access, needs, and access to services in a region.^1^,^2^

Despite the fact that global mortality rates have been decreasing over the past few decades, there is a focus on further reduction of under-five mortality in some nations.^4^ For example, under-five mortality rates ranged from 152 per 1000 live births in Guinea Bissau to 2.3 per 1000 live births in Singapore.^5^ In 2013, annualized rates of change from the year 1990 to 2013 ranged from −6.8% to 0.1%.^5^ The United Nations’ sustainable development goal (SDG) aims to reduce the under-five mortality in all countries to at least as low as 25 per 1,000 live births by 2030.^2^ According to the global statistics, over 5.6 million children, or 15,000 every day, died in this age group in 2016, making mortality under five years of age a major public health issue globally.^2^ Childhood mortality (defined as death between the first and the fifth birthday) was approximately 13.3 per 1000 live births globally in 2013.^5^ Childhood mortality in the North Africa/Middle East region and Qatar was approximately 5.4 and 2.1 per 1000 live births respectively in 2013.^5^ Generally, the sex ratio of male to female mortality aged between one and five years is greater than one, and females tend to have better survival up to five years when compared to males. In East Asian countries, there was an increase in sex ratio from 1.02 in 1990 to 1.14 in 2012.^6^

In this study, we aimed to investigate the trends in childhood mortality and the leading causes of death by nationality and sex in Qatar from 2004 to 2016. The mortality trends and characteristics among infants under one year of age in Qatar have already been discussed in our previous publication.^7^

## Methods

### Data Management and Handling

Ethical procedures as per Qatar Ministry of Public Health (MOPH) regulations were followed while carrying out the data handling and analysis. The data were maintained in a secure environment to assure privacy of personally identifiable information. Childhood mortality data were extracted from the MOPH Birth and Death Registry. Causes of death were coded in accordance with the International Classification of Diseases guidelines (ICD-10, Version 2016). The detailed process of data collection and “cause of death” coding was described elsewhere.^7^ The data contained information on date, place, and cause of death, as well as included demographic information like sex, age, and nationality (Qatari or non-Qatari).^7^

### Definition

The childhood mortality rate was defined as *“the probability of a child dying between the first and the fifth birthday, expressed as deaths per 1,000 children surviving to 12 months of age.”*^8^ This parameter is different than under-five mortality rate which also includes children who are under 1 year of age.^1^ An alternative approach to calculate childhood mortality (not used here) takes into account the number of deaths per 1000 live births, but the calculation method used in the present article is also acceptable and has been used globally in demographic health surveys.^5^, ^8^

### Statistical analysis

The sex ratio was calculated by dividing the mortality rates of males by that of the females. To assess the trends by sex ratio and each cause of childhood mortality, a Mann-Kendall trend test was performed. The Mann-Kendall trend test is a nonparametric test for monotonic trends in a time series, based on the Kendall rank correlation between the value of interest and time.^9^ MEDCALC^®^ was used to calculate the difference in the relative risk of the mortality due to selected causes by gender and nationality. All trend tests and plotting of changes in the mortality rates were carried out using R version 3.0.3 (R Foundation for Statistical Computing, Vienna, Austria). LOWESS (Locally Weighted Scatterplot Smoother) smoothing lines were added to the plots.

## Results

**Figure 1:**
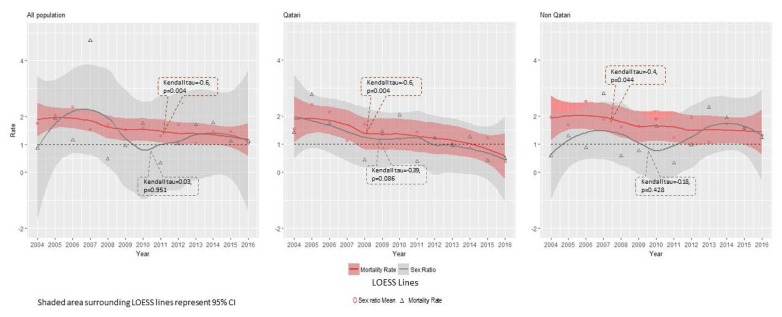
Annual rate and sex ratio of childhood mortality in Qatar by nationality

Overall, a total of 389 deaths were registered among children aged one to five in Qatar from 2004 to 2016 (Table 1). Figure 1 shows that the annual childhood mortality rates for all populations decreased significantly from 1.76 (Kendall tau=−0.6, p=0.004) in 2004 to 1.05 in 2016 (Kendall tau=−0.03, p=0.951), with an average rate of 1.6 per 1000 live births between these years (2004–2016). Based on the Kendall tau, this decrease was more significant for Qataris (Kendall tau=−0.6, p=0.004) than for Non-Qataris (Kendall tau=−0.4, p=0.044). However, there was no clear trend in sex ratio for the total study population (Kendall tau=0.03). We also found that the trend in sex ratio for Qatari population decreased from 1.41 in 2004 to 0.49 in 2016 but was not significant (Kendall tau=−0.39, p=0.086), and that it increased from 0.59 in 2004 to 1.24 in 2016 for non-Qataris but again was not significant (Kendall tau=−0.18, p=0.428).

**Figure 2:**
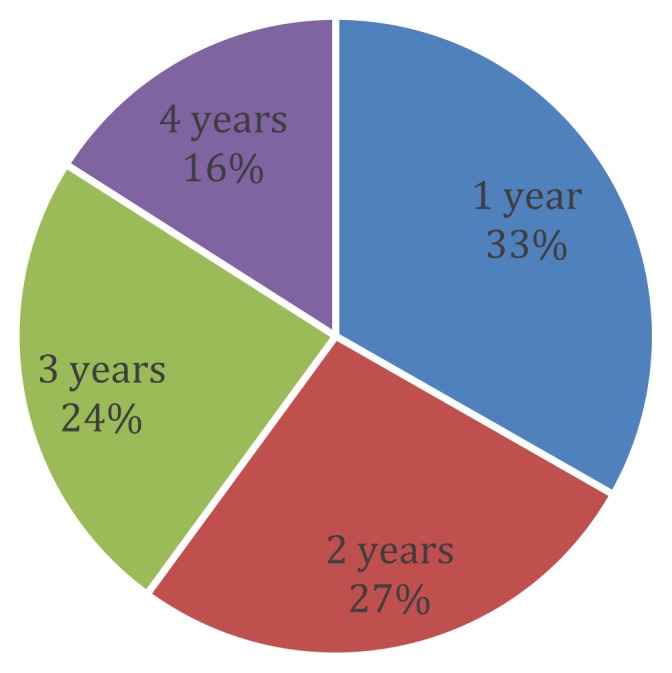
Proportion of Deaths by age for 2004–2016.

Overall (across all years), the majority of deaths occurred among children aged one (33%), followed by children aged two (27%), three (24%) and four (16%) (Figure 2).

Table 2 shows the trends in mortality rates across the top leading causes of mortality in Qatar by nationality. For the total population, even though transport accident-related mortality was prevalent, a decline was observed over time (Kendall tau= −0.03). Deaths in the “external cause of death” ICD category (not shown in the table) decreased (Kendall tau=−0.41, p-value = 0.06) as well. Furthermore, since 2004, the mortality due to congenital malformation of the circulatory system (Kendall tau=−0.36) and accidental drowning and submersion (Kendall tau=−0.54, p=0.012) also decreased.

By nationality, the Qatari population had a significant decrease in childhood mortality due to the congenital malformation of the circulatory system (Kendall tau=−0.67, p=0.003) and accidental drowning and submersion (Kendall tau=−0.55, p=0.016). Even though a decline in childhood mortality was seen among non-Qataris, the trends were not statistically significant (Table 2). The relative risk (RR) of death for Qataris versus Non-Qataris for the congenital malformations of the circulatory system was low (RR = 0.44, CI = 0.18 – 1.07, p = 0.07) (Table 3). A decline in transport accident-related mortality was observed among Qataris (Kendall tau= −0.14) and non-Qataris (Kendall= −0.12); however, this decline was not statistically significant (Table 2).

Table 4 shows the mean sex ratio and the trend of sex ratio by leading causes of death from 2004 to 2016. For the total population, the sex ratio was more than 1 for a majority of the causes of death and reached 2.75 for accidental drowning and submersion with a significant decrease in males over the observed period (Kendall tau=−0.72, p < 0.001). Qatari males had a higher relative risk of mortality due to accidental drowning and submersion in comparison to females (RR=7.77, CI = 0.97 – 62.15, p = 0.053) (data not shown).

Despite a significant decrease in the mortality trend for females (Kendall tau=−0.45, p=0.038) was observed, the sex ratio was found to be less than 1 (0.68) for congenital malformations of the circulatory system.

The Qatari population showed a higher mean sex ratio for congenital malformations of the circulatory system (Q20–Q28) and accidental drowning and submersion (W65–W74), which were 2.59 and 3.89 respectively. For congenital malformations of the circulatory system, the mortality trends significantly decreased for males (Kendall tau=−0.61, p=0.008), and not significantly for females (Kendall tau=−0.30). However, for accidental drowning and submersion there were no differences amongst both sexes for Qataris (Table 4). For accidental drowning and submersion, the mortality of the total population had a higher relative risk for males than females (RR = 2.75, CI= 1.16 – 6.50, p = 0.0212) (Table 3).

For the congenital malformations of the circulatory system among the non-Qatari population, the mean sex ratio was less than 1 with a significantly higher decline in mortality for females (Kendall tau=−0.45, p=0.038). Moreover, a higher mean sex ratio for malignant neoplasms was seen (2.23) but no significant change in the mortality trends between the years 2004 and 2016 was seen among both sexes. The sex ratio for diseases of the respiratory system was 0.72, with a significantly increasing trend among non-Qatari males from 2004 to 2016 (Kendall tau = 0.54, p = 0.031).

**Figure 3:**
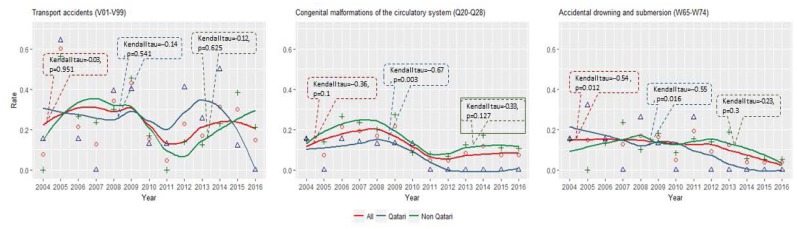
The distribution of the three major causes of child mortality from 2004 to 2016.

Figure 3 shows the distribution of the three major causes of childhood mortality from 2004 to 2016. While a declining trend in transport accidents was observed from 2004 to 2016, it was not significantly different for the total population and nationality subgroups.

The Qatari population showed a significant decrease in mortality rates for congenital malformations of the circulatory system (Kendall tau = −0.67, p = 0.003) and accidental drowning/submersion (Kendall tau= −0.55, p = 0.016). The latter was also significant for the total population (Kendall tau = −0.54, p = 0.012).

## Discussion

Based on the data from MOPH, this study described the trends in childhood mortality in the State of Qatar from 2004 to 2016. We found that the rates of mortality among children declined significantly from 1.76 per 1000 in 2004 to 1.05 per 1000 in 2016 (Figure 1). Globally, child mortality rates (per 1000 live births) reduced from 22.1 in 2000 to 13.1 in 2013 respectively.^5^ In 2013, the childhood mortality rates (per 1000 live births) were slightly higher in nearby Kuwait (1.8) and Oman (2.0) in comparison to Qatar (1.05); however, the rates were calculated using a different approach in our study and may not be comparable with other countries mentioned here.^5^ Moreover, the childhood mortality trends for the three leading causes of deaths have been decreasing over the past 12 years in Qatar. There was no clear trend of change in sex ratio found for the total study population. Moreover, the average childhood mortality rates by nationality (2004–2016) were 1.4 and 1.7 for Qataris and non-Qataris respectively, which are somewhat similar. This may be attributed to the universal healthcare access available in Qatar. The state offers a special health-card system for both non-nationals as well as nationals to obtain subsidized and, in some instances, free health services through a major public healthcare system.

Our study indicated that deaths due to external causes, including transport accident related deaths in Qatar, were similar to the findings from other countries such as the Republic of Korea, Japan, the United States, and the United Kingdom.^10^–^13^ A decreasing pattern in mortality due to external causes was also seen among these countries, similar to our findings in Qatar.^10^–^13^ A decline in transport accident related mortality was observed in the present study, and even though this trend was not statistically significant, it could be an early reflection of the effectiveness of programs implemented in Qatar. In 2007, a new law was passed in Qatar to control morbidity and mortality associated with road traffic accidents.^14^ The law included provisions for prohibiting children under 10 years of age to sit in the front seats of moving vehicles and also forbade the use of any portable devices such as mobile phones while driving.^14^ Furthermore, educational programs have been introduced in Qatar, including those targeting child passenger safety and promoting the use of a child/infant car seats.^15^ In addition, a National Road Safety Strategy 2013–2022 has been developed to decrease traffic accident related morbidity and mortality. The strategy multi-dimensionally targets traffic-related policies and infrastructure, as well as community education and awareness.^16^

The other two common causes of mortality discussed in this publication are accidental drowning/submersion and the congenital malformation of the circulatory system. A statistically significant decline from 2004 to 2016 was observed for accidental drowning and submersion for both the total population and Qataris. For the non-Qatari population, a non-significant decline was noticed. Among the Qatari population, the mortality due to the congenital malformations of the circulatory system has significantly decreased. For all three leading causes of mortality, the non-Qatari population had a non-significant decline. Mortality risk due to accidental drowning and submersion was higher among males compared with females for all national groups. The higher predisposition of drowning-related mortality among males has also been observed in other studies.^17^,^18^ However, in a Brazilian study, a slightly higher rate of childhood mortality due to malformations of the circulatory system was seen among females.^19^

Significant decreases were found for congenital malformations of the circulatory system among females in the total population, among Qatari males and non-Qatari females (Table 4). Furthermore, a significant decline in mortality due to accidental drowning and submersion was also seen among males in the total population (Table 4).

The major strength of this study was that the data came from a comprehensive birth and death registry system with a uniformly collected and accurate country-level childhood mortality information for 2004–2016. Data pertaining to the unknown causes of mortality is one of the challenges of our data collection system that may potentially impact mortality rates. The state of Qatar has a relatively stable Qatari citizens’ population compared to non-Qataris or expatriates. This can potentially lead to the selection bias in mortality indicators since expatriate population consists of relatively young healthy individuals.

Our results could be useful in supporting Qatar’s current national health strategy initiatives and also align with sustainable development goal that focus on ensuring healthy lives and promote well-being for citizens of all ages.^2^,^20^ These findings could also be essential for policymakers, researchers, and other professionals from regional countries as a reference or as a guideline tool. In summary, childhood mortality, though steadily decreasing, requires ongoing efforts and resources to be further reduced.

## Figures and Tables

**Table 1:** Childhood mortality rates by year, sex and nationality

Year	Children surviving to 12 months of age	One to five mortality	Childhood Mortality Rate				

	Count	Count	Total (per 1000)	Qatari	Non-Qatari	Male	Female
**2004**	13082	23	1.76	1.55	1.96	1.63	1.89
**2005**	13291	27	2.03	2.41	1.7	2.66	1.38
**2006**	14009	33	2.36	2.15	2.53	2.52	2.18
**2007**	15570	24	1.54	1.12	1.9	2.5	0.53
**2008**	17530	30	1.71	1.71	1.61	1.13	2.31
**2009**	18449	28	1.52	1.34	1.64	1.49	1.55
**2010**	19380	31	1.6	1.17	1.88	2.03	1.16
**2011**	20617	27	1.31	1.44	1.23	0.67	1.97
**2012**	21674	37	1.71	1.24	1.95	1.73	1.69
**2013**	23739	25	1.05	1.02	1.07	1.32	0.77
**2014**	25469	37	1.45	0.88	1.72	1.85	1.04
**2015**	26427	39	1.48	1.22	1.59	1.55	1.39
**2016**	26656	28	1.05	0.38	1.33	1.11	0.99

**Table 2:** The trends in the mortality rate across the top leading causes of child mortality by nationality

Population	Cause of death *(with ICD codes)*	Mean	Minimum		Maximum		Kendall tau	P value†

		2004–2016	Year	Mortality rate	Year	Mortality rate	2004–2016	2004–2016
Total	V01–V99 Transport accidents	0.24	2011	0.05	2005	0.60	−0.03	NS*
	R95–R99 Ill-defined and unknown causes of mortality	0.18	2009	0.11	2012	0.55	−0.01	NS
	Q20–Q28 Congenital malformations of the circulatory system	0.11	2012	0.05	2009	0.22	−0.36	NS
	W65–W74 Accidental drowning and submersion	0.11	2016	0.04	2011	0.19	−0.54	0.012
	C00–C97 Malignant neoplasms	0.05	2016	0.04	2004	0.15	−0.08	NS
	J95–J99 Other diseases of the respiratory system	0.05	2008	0.06	2015	0.19	0.10	NS
	G90–G99 Other disorders of the nervous system	0.04	2015	0.04	2008	0.17	−0.07	NS

Qatari	V01–V99 Transport accidents	0.25	2016	0.12	2005	0.64	−0.14	NS
	R95–R99 Ill-defined and unknown causes of mortality	0.13	2016	0.12	2006	0.46	−0.10	NS
	Q20–Q28 Congenital malformations of the circulatory system	0.06	2010	0.13	2004	0.16	−0.67	0.003
	W65–W74 Accidental drowning and submersion	0.09	2009	0.13	2005	0.32	−0.55	0.016
	C00–C97 Malignant neoplasms	0.04	2016	0.13	2012	0.27	0.10	NS
	J95–J99 Other diseases of the respiratory system	0.06	2016	0.13	2015	0.37	0.10	NS
	G90–G99 Other disorders of the nervous system	0.05	2013	0.13	2008	0.39	−0.14	NS

Non Qatari	V01–V99 Transport accidents	0.23	2013	0.13	2005	0.57	−0.12	NS
	R95–R99 Ill-defined and unknown causes of mortality	0.21	2008	0.10	2012	0.83	−0.01	NS
	Q20–Q28 Congenital malformations of the circulatory system	0.14	2012	0.07	2009	0.27	−0.33	NS
	W65–W74 Accidental drowning and submersion	0.11	2016	0.05	2007	0.24	−0.23	NS
	C00–C97 Malignant neoplasms	0.06	2012	0.07	2004	0.30	−0.10	NS
	J95–J99 Other diseases of the respiratory system	0.04	2008	0.10	2016	0.16	0.21	NS
	G90–G99 Other disorders of the nervous system	0.04	2015	0.05	2006	0.13	0.03	NS

† p-values based on Mann-Kendall trend test,

* NS – not significant

**Table 3:** Prominent causes of childhood mortality and relative risks by sex and nationality for 2004–2016

Cause of Death	Total Number of Deaths (2004–2016)	% of total Childhood Mortality (N=389)	Qatari Child Mortality Rate	Non-Qatari Child Mortality Rate	RR (95% CI)	Male CMR (n)	Female CMR (n)	RR (95% CI)
Overall	389	100%	1.32	1.63	0.81 (0.66 – 1.00)*	1.63	1.4	1.17 (0.95 – 1.42)
External Causes	127	33%	0.47	0.51	0.93 (0.64 – 1.33)	0.57	0.42	1.34 (0.94 – 1.91)
Transport Accidents	61	16%	0.25	0.23	1.33 (0.79 – 2.22)	0.28	0.20	1.39 (0.83 – 2.31)
Congenital malformations of the circulatory system	29	7%	0.06	0.14	0.44 (0.18 – 1.07)	0.09	0.14	0.68 (0.32 – 1.42)
Accidental drowning and submersion	27	7%	0.09	0.11	0.83 (0.37 – 1.86)	0.15	0.06	2.75 (1.16 – 6.50)†
Malignant neoplasms	14	4%	0.04	0.06	0.67 (0.21 – 2.13)	0.07	0.04	1.73 (0.58 – 5.17)

* p=0.0551,

† p=0.0212

**Table 4:** The sex ratio (males to females) for total mortality and for the leading causes of child mortality in Qatar (2004–2016)

Population	Cause of death (ICD code)	Mean	Kendall tau Males	P value	Kendall tau Females	P value†

		2004–2016	2004–2016	2004–2016	2004–2016	2004–2016
Total	V01–V99 Transport accidents	1.39	−0.13	NS	−0.04	NS
	R95–R99 Ill-defined and unknown causes of mortality	1.32	−0.09	NS	0.13	NS
	Q20–Q28 Congenital malformations of the circulatory system	0.68	−0.15	NS	−0.45	0.038
	W65–W74 Accidental drowning and submersion	2.75	−0.72	0.001	−0.16	NS
	C00–C97 Malignant neoplasms	1.73	0.04	NS	0.1	NS
	J95–J99 Other diseases of the respiratory system	1.12	0.21	NS	0.14	NS
	G90–G99 Other disorders of the nervous system	0.8	0.02	NS	−0.1	NS

Qatari	V01–V99 Transport accidents	1.15	−0.24	NS	0.04	NS
	R95–R99 Ill-defined and unknown causes of mortality	1.94	−0.07	NS	0.1	NS
	Q20–Q28 Congenital malformations of the circulatory system	2.59	−0.61	0.007	−0.3	NS
	W65–W74 Accidental drowning and submersion	3.89	0.07	NS	0.07	NS
	C00–C97 Malignant neoplasms	1.46	−0.41	NS	0.35	NS
	J95–J99 Other diseases of the respiratory system	0.97	−0.17	NS	0.33	NS
	G90–G99 Other disorders of the nervous system	0.65	0.02	NS	−0.26	NS

Non Qatari	V01–V99 Transport accidents	1.57	0.01	NS	−0.01	NS
	R95–R99 Ill-defined and unknown causes of mortality	1.15	0.03	NS	0.1	NS
	Q20–Q28 Congenital malformations of the circulatory system	0.62	−0.05	NS	−0.45	0.038
	W65–W74 Accidental drowning and submersion	1.91	−0.3	NS	−0.1	NS
	C00–C97 Malignant neoplasms	2.23	0.16	NS	−0.12	NS
	J95–J99 Other diseases of the respiratory system	0.72	0.54	0.031	0.07	NS
	G90–G99 Other disorders of the nervous system	0.96	0.12	NS	0.02	NS

† p-values based on Mann-Kendall trend test,

* NS – not significant
